# The PorX Response Regulator of the *Porphyromonas gingivalis* PorXY Two-Component System Does Not Directly Regulate the Type IX Secretion Genes but Binds the PorL Subunit

**DOI:** 10.3389/fcimb.2016.00096

**Published:** 2016-08-31

**Authors:** Maxence S. Vincent, Eric Durand, Eric Cascales

**Affiliations:** Laboratoire d'Ingénierie des Systèmes Macromoléculaires, Institut de Microbiologie de la Méditerranée, Aix-Marseille Université – Centre National de la Recherche Scientifique (UMR7255)Marseille, France

**Keywords:** transcriptional regulation, type IX secretion, gingivitis, periodontitis, *Porphyromonas gingivalis*, two-component system, bacterial pathogenesis

## Abstract

The Type IX secretion system (T9SS) is a versatile multi-protein complex restricted to bacteria of the *Bacteriodetes* phylum and responsible for the secretion or cell surface exposition of diverse proteins that participate to S-layer formation, gliding motility or pathogenesis. The T9SS is poorly characterized but a number of proteins involved in the assembly of the secretion apparatus in the oral pathogen *Porphyromonas gingivalis* have been identified based on genome substractive analyses. Among these proteins, PorY, and PorX encode typical two-component system (TCS) sensor and CheY-like response regulator respectively. Although the *porX* and *porY* genes do not localize at the same genetic locus, it has been proposed that PorXY form a *bona fide* TCS. Deletion of *porX* in *P. gingivalis* causes a slight decrease of the expression of a number of other T9SS genes, including *sov, porT, porP, porK, porL, porM, porN*, and *porY*. Here, we show that PorX and the soluble cytoplasmic domain of PorY interact. Using electrophoretic mobility shift, DNA-protein co-purification and heterologous host expression assays, we demonstrate that PorX does not bind T9SS gene promoters and does not directly regulate expression of the T9SS genes. Finally, we show that PorX interacts with the cytoplasmic domain of PorL, a component of the T9SS membrane core complex and propose that the CheY-like PorX protein might be involved in the dynamics of the T9SS.

## Introduction

Periodontitis and gum diseases are considered as major public health concerns as a recent prevalence study of the Centers for Disease Control and Prevention reported that roughly 47% of the USA adults aged 30 years or older suffer of serious tooth tissue damages and tooth decay (Eke et al., 2012). These diseases are due to a lack of dental hygiene and by the action of deleterious bacteria. The major bacterium responsible for periodontitis, gingivitis, and other gum diseases is *Porphyromonas gingivalis* (Kamma et al., 1994; Lo Bue et al., 1999; Hussain et al., 2015). *P. gingivalis* is an anaerobic bacterial oral pathogen that causes severe lesions in periodontal tissues such as the gingiva or the alveolar bone by disrupting the tooth-supporting structure (Bostanci and Belibasakis, 2012). In addition, recent studies reported links between periodontitis and systemic health issues, such as higher risks of cardiovascular diseases or rheumatoid arthritis (Janssen et al., 2013; Koziel et al., 2014). Rheumatoid arthritis is caused by the citrullination activity of a specific enzyme, the peptidylarginine deiminase (PPAD; Koziel et al., 2014; Gabarrini et al., 2015). By contrast, tissue damages are mainly induced by a cocktail of specialized toxin proteins secreted by the bacterium, collectively known as gingipains (Fitzpatrick et al., 2009). Gingipains act as adhesins or proteases that help the bacterium to adhere to periodontal tissues and to promote gingival tissue invasion by the degradation of matrix proteins such as fibrinogen and collagen (Fitzpatrick et al., 2009; Nakayama, 2015). The active release of gingipains at the bacterial cell surface and of the PPAD is catalyzed by a multi-protein complex, the Type IX secretion system (T9SS, Sato et al., 2010; Nakayama, 2015). Eleven genes, called *por*, have been identified in the *P. gingivalis* strain ATCC 33277 using substractive genome analyses, mutagenesis, and proteomic studies and have been shown to be involved in gingipain and PPAD transport to the cell surface (Sato et al., 2010, 2013; Taguchi et al., 2015; Gorasia et al., 2016). It is therefore thought that these 11 subunits assemble a trans-envelope channel that specifically recruits the toxins and transports them to the cell surface. A complex composed of the PorK, PorL, PorM, and PorN proteins has been isolated and visualized by blue-native gel electrophoresis (Sato et al., 2010). However, the T9SS is not restricted to *P. gingivalis* strains. A number of studies have reported the presence of T9SS genes in bacteria of the *Bacteriodetes* phylum, including species of the *Flavobacterium, Cytophaga, Cellulophaga, Capnocytophaga*, or *Tannerella* genus (McBride and Zhu, 2013). In these strains, the T9SS is responsible for the cell surface exposition, attachment, or external release of very diverse proteins (Sato et al., 2010; Shrivastava et al., 2013; Narita et al., 2014; Tomek et al., 2014; Zhu and McBride, 2014; Kita et al., 2016). This machine has been therefore adapted to the specific needs of each bacterium. In *T. forsythia*, the T9SS is necessary for the transport of the components of the outmost S-layer (Narita et al., 2014; Tomek et al., 2014). In *F. johnsioniae*, the T9SS is responsible for the transport and propulsion of adhesins at the cell surface allowing the bacterium to glide on solid surfaces (Nakane et al., 2013; Shrivastava et al., 2013; McBride and Nakane, 2015). The *F. johnsoniae* adhesins are rotative filaments (Nakane et al., 2013; Shrivastava et al., 2015). Similarly to the flagellum, the T9SS is thought to act as a proton-motive force-dependent trans-envelope motor and to power the rotation of the adhesins (Nakane et al., 2013; McBride and Nakane, 2015; Shrivastava and Berg, 2015; Shrivastava et al., 2015).

In addition to structural subunits of the transport apparatus, the substractive analyses performed with *P. gingivalis* revealed two additional genes, *porY* and *porX* (Sato et al., 2010). These two genes encode a two-component sensor and response regulator respectively. Although the two genes are not encoded within a single genetic unit, it has been proposed that these two proteins form a two-component system responsible for regulation of the *por* genes. Indeed, microarray analyses showed that PorX and PorY contribute to the regulation of the T9SS *por* genes as a 1.8-fold decrease of their expression is observed in *porX* and *porY* mutant cells compared to the WT strain (Sato et al., 2010). Here, we build up on this result and show that PorX and PorY interact and likely constitute a *bona fide* two-component system. We did not detect binding of PorX to the promoter regions of the *sov, porT*, and *porP* genes, and we did not observe increased activity of this promoters in presence of PorX suggesting that PorX does not directly regulate these genes. Indeed, domain analyses of PorX did not reveal any DNA binding motif, but rather a CheY-like receiver domain. We further show that PorX binds to the cytoplasmic domain of the T9SS PorL proteins, and that a specific patch of hydrophobic residues of PorL mediate this interaction.

## Materials and methods

### Bacterial strains, plasmids, medium, and growth conditions

Strains and plasmids used in this study are listed in the Supplemental Table. The *P. gingivalis* ATCC33277 (DSM-20709) wild-type strain used in this study was obtained from the German collection of microorganisms and cell cultures (DSMZ). The DH5α, BTH101, W3110, and BL21(DE3) *E. coli* K-12 strains were used for cloning, bacterial two-hybrid, co-immune-precipitation, and protein production and purification respectively. *E. coli* cells were routinely grown in Luria Broth (LB) supplemented with antibiotics when necessary (kanamycin 50 μg/mL, ampicillin 100 μg/mL, chloramphenicol 30 μg/mL). *P. gingivalis* cells were grown anaerobically in Brain Heart Infusion medium supplemented with hemin (5 μg/mL) and menadione (0.5 μg/mL) in Hungate tubes.

### Plasmid construction

Polymerase Chain Reactions (PCR) were performed using a Biometra thermocycler using the Q5 (New England Biolabs) or Pfu Turbo (Agilent Technologies) DNA polymerases. Restriction enzymes were purchased from New England Biolabs and used according to the manufacturer's instructions. Custom oligonucleotides were synthesized by Sigma Aldrich and are listed in the Supplemental Table. *P. gingivalis* chromosomal DNA was used as a template for PCRs. Bacterial two-hybrid vectors have been constructed by ligation of *Eco*RI-*Bam*HI fragments into the vectors pUT18C and pKT25 (Karimova et al., 1998). Cloning of *porX* into the pETG20A vector was performed according to standard Gateway protocols. Other DNA fragment insertions into the pBAD24 (Guzman et al., 1995) and pUA66 (Zaslaver et al., 2006) vectors have been performed by restriction-free cloning (van den Ent and Löwe, 2006). Briefly, the gene of interest was amplified using oligonucleotides introducing extensions annealing to the target vector. The double-stranded product of the first PCR has then been used as oligonucleotides for a second PCR using the target vector as template. PCR products were then treated with *Dpn*I to eliminate template plasmids and transformed into DH5α-competent cells. All constructs have been verified by restriction analyses and DNA sequencing (Eurofins or MWG). The expression of the cloned genes or fragments was induced with IPTG (0.5 mM), L-arabinose (0.02%), or anhydrotetracycline (AHT, 0.02 μg/mL).

### Bacterial two-hybrid (bacth)

The adenylate cyclase-based bacterial two-hybrid technique (Karimova et al., 1998) was used as previously published (Battesti and Bouveret, 2008, 2012; Zoued et al., 2013). Briefly, the proteins to be tested were fused to the isolated T18 and T25 catalytic domains of the *Bordetella* adenylate cyclase. After introduction of the two plasmids producing the fusion proteins into the reporter BTH101 strain, plates were incubated at 30°C for 48 h. Three independent colonies for each transformation were inoculated into 600 μL of LB medium supplemented with ampicillin, kanamycin and IPTG (0.5 mM). After overnight growth at 30°C, 10 μL of each culture were dropped onto LB plates supplemented with ampicillin, kanamycin, IPTG, and X-Gal and incubated for 16 h at 30°C. The experiments were done at least in triplicate and a representative result is shown.

### Co-immune-precipitation

100 mL of *E. coli* W3110 cells bearing the plasmid of interest were grown to an optical density at λ = 600 nm (*A*_600_) ~ 0.4 and the expression of the cloned genes were induced with AHT or L-arabinose for 45 min. The cells were harvested, and the pellets were resuspended in Tris-HCl 20 mM pH 8.0, NaCl 100 mM, sucrose 30%, EDTA 1 mM, lysozyme 100 μg/mL, DNase 100 μg/mL, RNase 100 μg/mL supplemented with protease inhibitors (Complete, Roche) to an *A*_600_ of 80, incubated on ice for 20 min and cells were lysed by three passages at the French Press (800 psi). The lysates were clarified by centrifugation at 20,000 × g for 20 min. and the supernatants containing the soluble proteins were used for co-immunoprecipitation using anti-FLAG M2 affinity gel (Sigma-Aldrich). After 3 h of incubation on a wheel, the beads were washed three times with 1 mL of Tris-HCl 20 mM pH 8.0, NaCl 100 mM, sucrose 15%, air-dried, resuspended in 25 μL of Laemmli loading buffer, boiled for 10 min, and subjected to SDS-PAGE and immunodetection analyses.

### Protein purification

*E. coli* BL21(DE3) cells carrying pETG20A-PorX or pRSF-PorY_C_ were grown at 37°C in LB to an *A*_600_ ~ 0.7 and gene expression was inducted with 0.5 mM IPTG for 16 h at 22°C. Cells were harvested, resuspended in Tris-HCl 50 mM pH 8.0, NaCl 300 mM supplemented with lysozyme (100 μg/mL), DNase (100 μg/mL), MgCl_2_ 5 mM, and imidazole 10 mM, and broken by three passages at the French press (800 psi). Soluble proteins were separated from inclusion bodies and cell debris by centrifugation 30 min at 16,000 × g. The His-tagged fusions were purified using ion metal Ni^2+^ affinity chromatography (IMAC) using a 5-mL HisTrap column (GE Healthcare) and eluted with a step gradient of imidazole. For PorY_C_, the collected fractions were analyzed by SDS-PAGE and Coomassie blue staining and the fractions of interest were pooled and dialized for 16 h at 4°C in Tris-HCl 50 mM pH 8.0, NaCl 150 mM. The final concentration of PorY_C_ was 1.23 mg/mL. The PorX protein, purified from the pETG20A vector, is fused to a Tobacco Etch Virus (TEV) protease-cleavable thioredoxin-6 × His tag. After the first IMAC, the fusion PorX proteins were digested overnight at 4°C by a 6 × His-tagged TEV protease using a 1:10 (w/w) protease:protein ratio. The TEV protease and contaminants were retained by a second IMAC and the purified proteins were collected in the flow through. Proteins were further separated on preparative Superdex 200 or Superose 6 gel filtration column (GE Healthcare) equilibrated in Tris-HCl 20 mM pH 8.0, NaCl 150 mM. The fractions containing the purified protein were pooled and concentrated by ultrafiltration using the Amicon technology (Millipore, California, USA). The final concentration of PorX was 11.6 mg/mL. The *E. coli* Ferric uptake regulator (Fur) and sequence signal-less Peptidoglycan-associated lipoprotein (Pal) were purified as previously described (Abergel et al., 2001; Brunet et al., 2011).

### Protein-protein co-purification

The purified PorX and 6 × His-tagged PorY_C_ or Pal proteins were mixed and incubated on ice for 10 min. The mixture was then applied to Co^2+^-NTA beads (Talon, Clontech) in Tris-HCl 50 mM pH 8.0, NaCl 150 mM and incubated at 25°C for 45 min. The beads were washed three times with Tris-HCl 50 mM pH 8.0, NaCl 150 mM supplemented with imidazole 10 mM, air-dried, resuspended in 25 μL of Laemmli loading buffer, boiled for 10 min, and subjected to SDS-PAGE and immunodetection analyses.

### DNA-protein co-purification

PCR fragments were obtained using a biotinylated 5′ oligonucleotide, and immobilized on streptavidin beads (DyNAbeads, Lifescience) in Tris-HCl 10 mM pH 7.5, EDTA 0.5 mM, NaCl 1 mM. Unbound biotinylated probes were eliminated by three washes in phosphate-buffered saline (PBS). The purified PorX and Fur proteins were mixed, and the mixture was then applied to the DNA-coated streptavidin beads in PBS at 25°C for 45 min on a wheel. The beads were washed three times in PBS, air-dried, resuspended in 25 μL of Laemmli loading buffer, boiled for 10 min, and subjected to SDS-PAGE and immunodetection analyses.

### Electrophoretic mobility shift assay

PCR products were purified using the Wizard Gel and PCR clean-up kit (Promega). 250 ng of PCR products were incubated in a final volume of 10 μl in Tris-HCl 40 mM pH 7.2, NaCl 50 mM, KCl 5 mM, Glycerol 5%, MgCl_2_ 6 mM, CaCl_2_ 0.5 mM, acetylphosphate 10 mM, dithiothreitol 1 mM, bovine serum albumine 100 μg/mL in presence of purified PorX (0, 50, 100, 200, or 400 nM). After incubation for 25 min at 25°C, the samples were loaded on a pre-run 8% non denaturing polyacrylamide (Tris-borate) gel, and DNA and DNA-complexes were separated at 100 V in Tris-Borate buffer (45 mM Tris base, 45 mM boric acid, 100 μM MnCl_2_).

### Heterologous host regulation assay

W3110 cells carrying the empty vector pBAD24 or pBAD-PorX and the promoter-pUA66 derivatives were grown in 96-well microplates to an *A*_600nm_ ~ 0.4, treated with L-arabinose for 45 min, and the *A*_600nm_ and the levels of fluorescence were acquired at 505 nm after excitation at 488 nm using a TECAN microplate reader. Reported values represent the average of technical triplicates from three independent biological cultures and standard deviation are shown on the graphs.

### Computer analyses

Domain homology analyses were performed with pfam (Finn et al., 2016; http://pfam.xfam.org/), HHpred (Söding et al., 2005; http://toolkit.tuebingen.mpg.de/hhpred), and Phyre^2^ (Kelley et al., 2015; http://www.sbg.bio.ic.ac.uk/phyre2). Predictions of trans-membrane domains were performed with TMPred (Hofmann and Stoffel, 1993; http://www.ch.embnet.org/software/TMPRED_form.html), TMHMM (Sonnhammer et al., 1998; http://www.cbs.dtu.dk/services/TMHMM/), and HHMTop (Tusnády and Simon, 1998; http://sbcb.bioch.ox.ac.uk/TM_noj/TM_noj.html).

### Miscellaneous

SDS-polyacrylamide gel electrophoresis was performed using standard protocols. For immunostaining, proteins were transferred onto nitrocellulose membranes, and immunoblots were probed with primary antibodies and goat secondary antibodies coupled to alkaline phosphatase, and developed in alkaline buffer in presence of 5-bromo-4-chloro-3-indolylphosphate and nitroblue tetrazolium. The anti-FLAG (M2 clone, Sigma-Aldrich), anti-VSV-G (P5D4 clone, Sigma-Aldrich) monoclonal antibodies, and alkaline phosphatase-conjugated goat anti-mouse secondary antibodies (Beckman Coulter) have been purchased as indicated and used as recommended by the manufacturers.

## Results and discussion

### Sequence analyses of PorX and PorY

**Figure 1:**
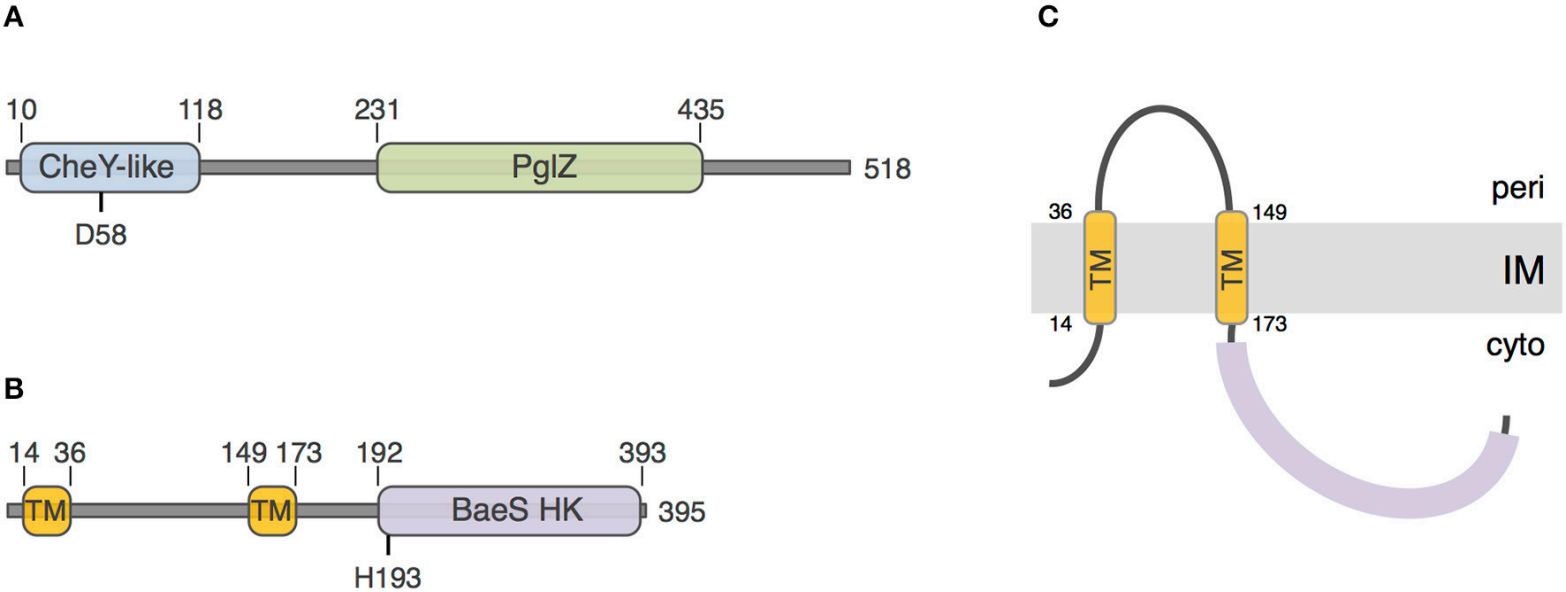
**Sequence analyses of PorX and PorY**. Schematic representation of the PorX **(A)** and PorY **(B)** proteins. The number of amino-acids of the protein is indicated on the right. The predicted receiver CheY-like, PglZ, and BaeS histidine kinase (BaeS HK) domains and transmembrane helices (TM) are indicated, as well as their boundaries. The putative residues involved in phospho-transfer [Aspartate-58 (D58) of PorX and Histidine-193 (H193) of PorY] are indicated. **(C)** Predicted topology of the PorY protein at the inner membrane (IM).

To gain insights onto the PorX and PorY proteins, we performed a sequence and structural analysis using the HHPred and Phyre^2^ algorithms. The 518-aminoacid PorX protein [PGN_1019, gene accession (GI): 188594563] is a putative cytoplasmic protein that is constituted of two domains: a N-terminal receiver domain of the CheY family (pfam identifier: PF00072) and a C-terminal effector domain of the PglZ family (pfam: PF08665) (Figure 1A). CheY is a phospho-protein involved in chemotaxis, coupling signal sensing via chemoreceptors to the control of the directionality of the flagellar rotation (Sourjik and Wingreen, 2012). This regulation occurs via the phosphorylation of a conserved aspartate residue that is also present in PorX, at position 58 (Figure 1A). The function of PglZ domains is still unknown but these domains do not bear typical motif responsible for DNA binding. The 395-aminoacid PorY protein (PGN_2001, GI: 188595544) is predicted to be anchored to the inner membrane via two trans-membrane segments located between residues 14 and 36, and 149 and 173, delimitating a ~115-aminoacid periplasmic loop probably involved in signal detection (Figures 1B,C). The PorY cytoplasmic domain is anticipated to adopt a classical signal transduction histidine kinase fold of the BaeS family with an ATP binding site and a phospho-acceptor domain that bears a conserved histidine residue at position 193 (Figure 1B).

### PorY and PorX constitute a *bona fide* two-component system

**Figure 2:**
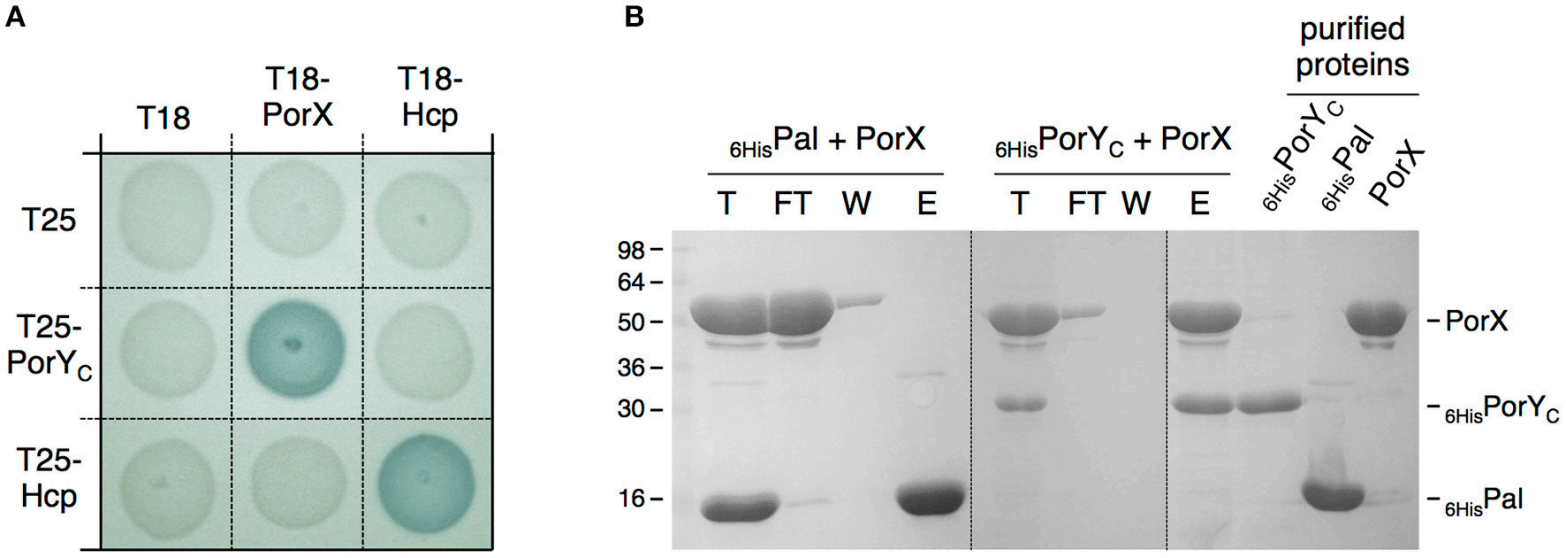
**PorX and PorY interact**. **(A)** Bacterial two-hybrid assay. BTH101 reporter cells carrying pairs of plasmids producing the indicated proteins fused to the T18 or T25 domain of the *Bordetella* adenylate cyclase were spotted on X-Gal-IPTG reporter LB agar plates. Controls include T18 and T25 fusions to Hcp (Zoued et al., 2013), a T6SS protein from enteroaggregative *E. coli* and unrelated to the T9SS. **(B)** Co-purification assay. The indicated proteins (individually shown on the right) were mixed, and the total sample (T) was incubated with Ni-NTA beads. Unbound [flow through (FT) and wash (W)] and bound (E) proteins were collected and analyzed by SDS-PAGE and Coomassie blue staining. The different proteins are indicated on the right. Molecular weight markers (in kDa) are indicated on the left.

The *porY* and *porX* genes are separated by ~1000 genes on the *P. gingivalis* chromosome, a genetic organization that is uncommon for cognate TCS, which are usually arranged as dicistronic units. This organization raised the question whether these two proteins are partners. To test the interaction between PorX and PorY, we performed bacterial two-hybrid experiments. The PorX protein was fused to the T18 domain, and its interaction with the cytoplasmic soluble domain of PorY (PorY_C_) fused to the T25 domain was assayed on reporter plates. Figure 2A shows that the PorX-PorY_C_ interaction reconstitutes the activity of the adenylate cyclase. This interaction was confirmed by co-purification. PorX and PorY_C_ were first purified to homogeneity. Figure 2B shows that the immobilization of the 6 × His-tagged PorY_C_ protein—but not that of the 6 × His-tagged Pal unrelated control protein—on the Ni-NTA affinity resin retains the untagged PorX protein. Taken together, these results demonstrate that PorY and PorX interact and likely constitute a *bona fide* TCS. This result is consistent with the observation that the deletions of the *porX* and *porY* genes have similar effects on T9SS gene expression (Sato et al., 2010).

### PorX is not a direct regulator of T9SS genes

**Figure 3:**
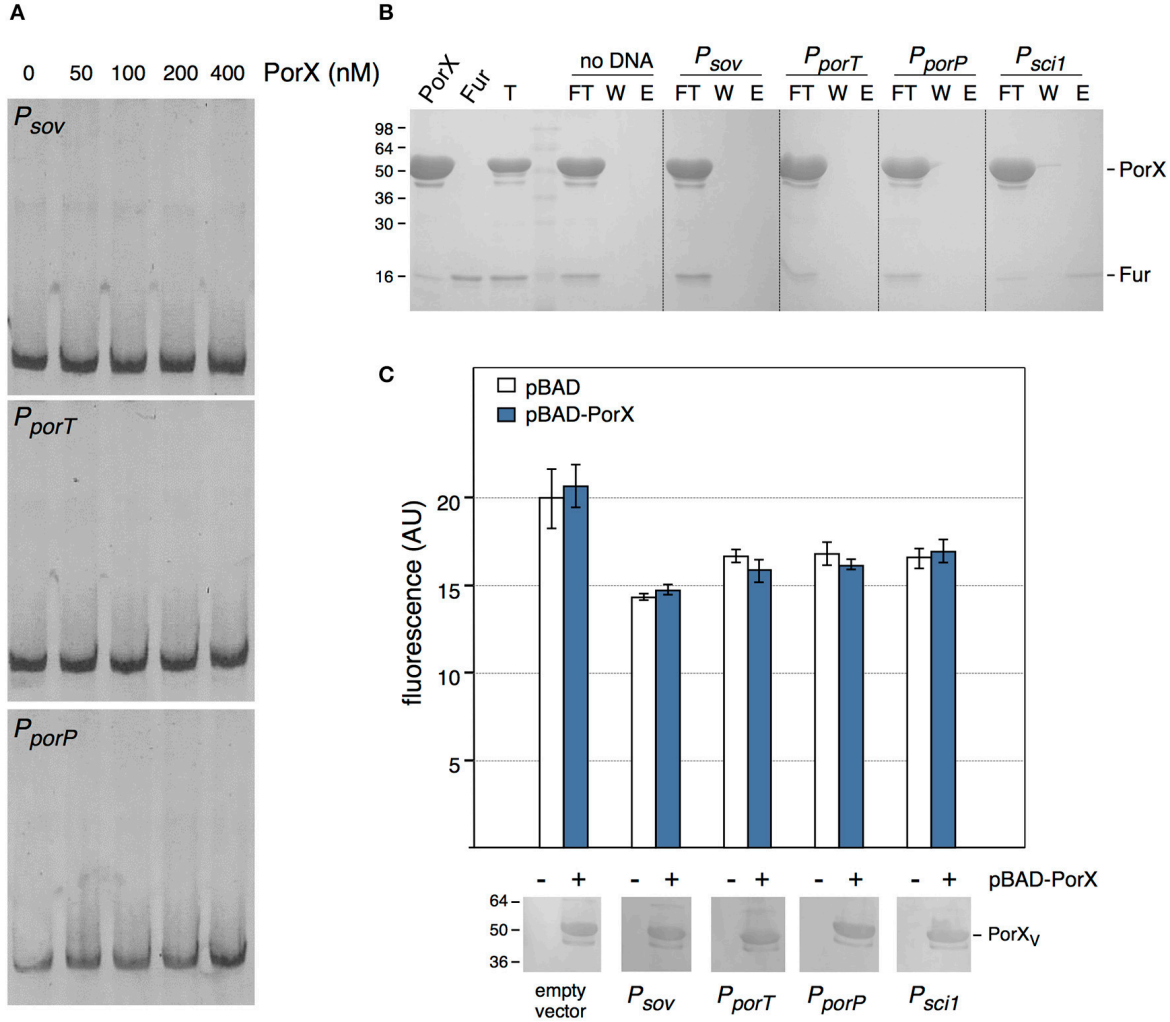
**PorX is not a direct regulator of T9SS genes. (A)** Electrophoretic mobility shift assay (EMSA) of the *sov* (*P*_*sov*_), *porT* (*P*_*porT*_), or *porP* (*P*_*porP*_) promoter with the indicated concentrations of the purified PorX protein. **(B)** DNA-protein co-purification assay. The purified PorX and Fur proteins (individually shown on the left) were mixed, and the total sample (T) was incubated with streptavidin beads coated with the indicated biotinylated promoter fragment (no DNA, streptavidin beads alone; *P*_*sci*1**,_ promoter fragment of the Fur-regulated enteroaggregative *E. coli sci1* T6SS gene cluster). Unbound [flow through (FT) and wash (W)] and bound (E) proteins were collected and analyzed by SDS-PAGE and Coomassie blue staining. The different proteins are indicated on the right. Molecular weight markers (in kDa) are indicated on the left. **(C)** Promoter/regulator reconstitution assay in the *E. coli* heterologous host. Relative fluorescence levels (in arbitrary units) of *E. coli* cells carrying the indicated pUA66-promoter derivatives (GFP under the control of the indicated promoter) were measured in absence (−, white bars) or presence (+, blue bars) or PorX. Is represented the mean of fluorescence levels obtained from three independent experiments (each measured in triplicate). The production of VSV-G-tagged PorX is shown on bottom (immunodetection with anti-VSV-G monoclonal antibody; molecular weight markers on left).

We then asked whether PorX regulates directly the T9SS genes by binding to their promoter regions. Sequence analyses of the promoter sequences of a subset of *por* genes highlighted a conserved palindromic motif that may constitute a binding box for a transcriptional regulator. 400-bp DNA fragments encompassing the promoter regions of *sov* (−373 to +33 relative to the putative +1 transcriptional start site), *porT* (−413 to +60) and of the *porPKLMN* operon (−309 to +34) were amplified and used for electrophoretic mobility shift assays (EMSA) with the purified PorX protein. We did not observe retardation of these DNA probes with up to 400 nM of PorX (Figure 3A), suggesting that no binding occurs. To test *por* promoters-PorX interaction by an alternative approach, the promoters were biotinylated, incubated with a mixture of PorX and of the *E. coli* Fur protein and then immobilized on a streptavidin-loaded resin. Figure 3B shows that the *sov, porT*, and *porP* promoters did not retain PorX whereas Fur was specifically retained by the Fur-dependent enteroaggregative *E. coli* T6SS *sci1* promoter (Brunet et al., 2011). These results therefore suggest that PorX does not bind to the *por* gene promoters. This conclusion was strengthened by a promoter/PorX reconstitution approach in the *E. coli* K-12 heterologous host. Translational reporter fusions between the promoters and the GFP-encoding gene were engineered and the expression of the *gfp* (under the control of the cloned fragments) was followed at 505 nm (Figure 3C). In absence of PorX, the levels of fluorescence of the reporter fusions were similar to that of the parental promoterless pUA66 vector or the Fur-repressed pUA66-*sci1* construct, suggesting that the *por* genes are not expressed (Figure 3C, white bars). In presence of PorX, the levels of fluorescence of the reporter fusions were comparable to that in absence of PorX (Figure 3C, blue bars), demonstrating that PorX does not increase the activity of the promoters, and that these promoters are not under the direct control of PorX. This result is in agreement with the observation that PorX lacks DNA binding motifs.

### The CheY-like PorX protein interacts with the cytoplasmic domain of the PorL T9SS membrane core subunit

**Figure 4:**
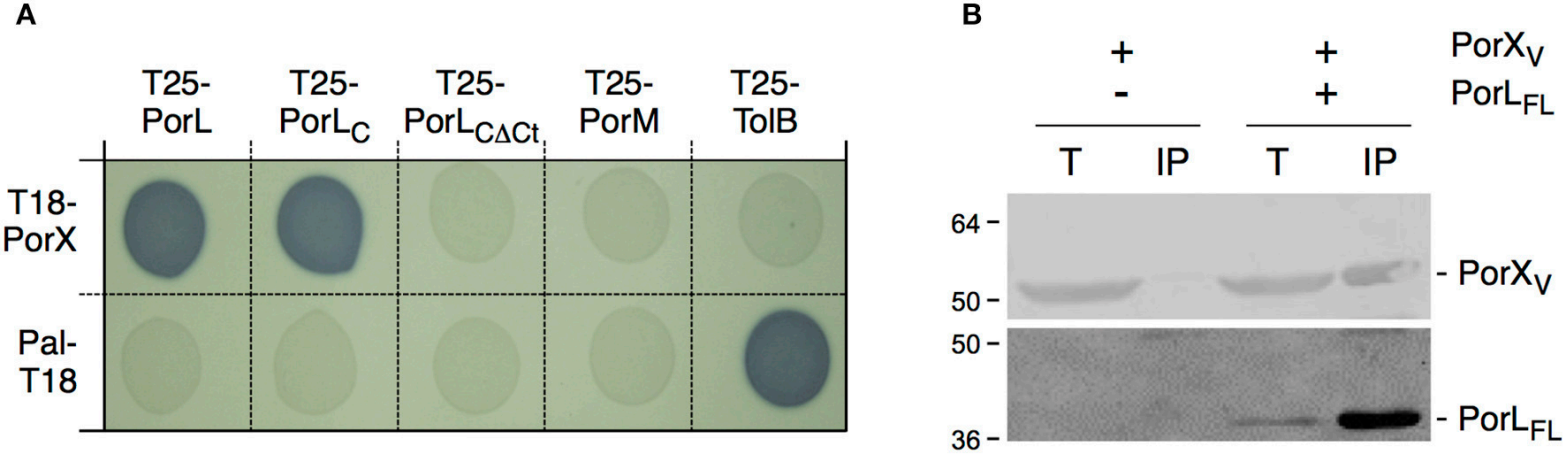
**PorX interacts with the cytoplasmic domain of the PorL T9SS subunit. (A)** Bacterial two-hybrid assay. BTH101 reporter cells carrying pairs of plasmids producing the indicated proteins fused to the T18 or T25 domain of the *Bordetella* adenylate cyclase were spotted on X-Gal-IPTG reporter LB agar plates. Controls include T18 and T25 fusions to Pal and TolB, two proteins unrelated to the T9SS. **(B)** Co-immune precipitation assay. Lysates producing the indicated proteins were mixed and subjected to immune precipitation with anti-FLAG antibodies. The total input (T) and immunoprecipitated (IP) material were analyzed by SDS-PAGE and immuno-detected with anti VSV-G (upper panel) and anti-FLAG (lower panel) monoclonal antibodies. The proteins are indicated on the right. The molecular weight markers are indicated on the left.

The fact that PorX is not a direct regulator of T9SS genes prompted us to investigate further how PorX could influence the T9SS. The initial sequence analyses defined that the N-terminal domain of PorX is similar to response regulators of the CheY family, which are involved in chemotaxis. Once phosphorylated, CheY binds to the flagellar C-ring (Roman et al., 1992; Sagi et al., 2003), and more specifically to the FliM and FliN switch complex proteins (Shukla et al., 1998; Paul et al., 2006; Sarkar et al., 2010) hence reversing the direction of rotation of the filament. Binding of CheY to FliN is mediated by an hydrophobic patch within FliN (Paul et al., 2006; Sarkar et al., 2010). Interestingly, it has been recently reported that the T9SS is also a rotary machine, and specifically that it promotes rotary movement of the SprB filamentous adhesin at the cell surface of *F. johnsioniae* (Shrivastava and Berg, 2015). We therefore asked whether, similarly to CheY, PorX is recruited to one of the basal components of the T9SS. The T9SS subunits are poorly characterized but the system is principally constituted of outer membrane-associated proteins (McBride and Zhu, 2013; McBride and Nakane, 2015; Nakayama, 2015; Gorasia et al., 2016). However, two proteins, PorL and PorM, are anchored to the inner membrane and have therefore cytoplasmic regions or domains (Sato et al., 2010). We therefore tested whether PorX contacts PorL and/or PorM. Bacterial two-hybrid experiments revealed that PorX and PorL interact whereas no interaction was detected between PorX and PorM (Figure 4A). In addition, we showed that PorX forms a precipitable complex with the cytoplasmic domain of PorL (PorL_C_ (amino-acids 73–309), Figures 4A,B). Interestingly, the cytoplasmic domain of PorL carries a hydrophobic patch near its C-terminus (residues 279–298; LLQALTTNVGLPGMPGNFGA). Deletion of this hydrophobic region [PorL_C△Ct_ (amino-acids 73–274)] abolished the interaction with PorX (Figure 4A). We therefore conclude that PorX is recruited to the basal part of the T9SS core apparatus via direct interaction with an hydrophobic patch of PorL, a situation reminiscent to the CheY recruitment to the flagellar switch complex.

### Concluding remarks

Sequence analyses of the *P. gingivalis* PorX and PorY proteins showed that PorX is composed of a CheY-like receiver domain and a PglZ domain of unknown function, whereas the cytoplasmic histidine kinase domain of PorY is anchored to the inner membrane through a trans-membrane hairpin. This prediction is in agreement with recent results published by Kadowaki et al. who showed that PorY co-fractionates with the inner membrane fraction (Kadowaki et al., 2016). Using bacterial two-hybrid and co-purification experiments, we then demonstrate that PorY and PorX interact and likely assemble a cognate signal sensor/response regulator pair. Our results are in agreement with surface plasmon resonance studies that revealed that the two proteins interact with a K_*D*_ of 1.41 μM and that PorY is capable of phospho-transfer onto PorX (Kadowaki et al., 2016). Using electrophoretic mobility shift and DNA-co-purification assays, we showed that PorX does not bind to the promoter regions of three T9SS genes or operons, *sov, porT*, and *porP*, suggesting that PorX does not directly regulate these genes. This hypothesis was validated by promoter/PorX reconstitution experiments in the *E. coli* heterologous host that showed that the presence of PorX does not increase the activity of these promoters. Kadowaki et al. recently identified a direct regulator of T9SS genes, SigP (Kadowaki et al., 2016). SigP is a transcriptional regulator of the ECF sigma factor family that binds and activates the expression of the T9SS genes. The authors nicely showed that PorX interacts and stabilizes SigP (Kadowaki et al., 2016).

**Figure 5:**
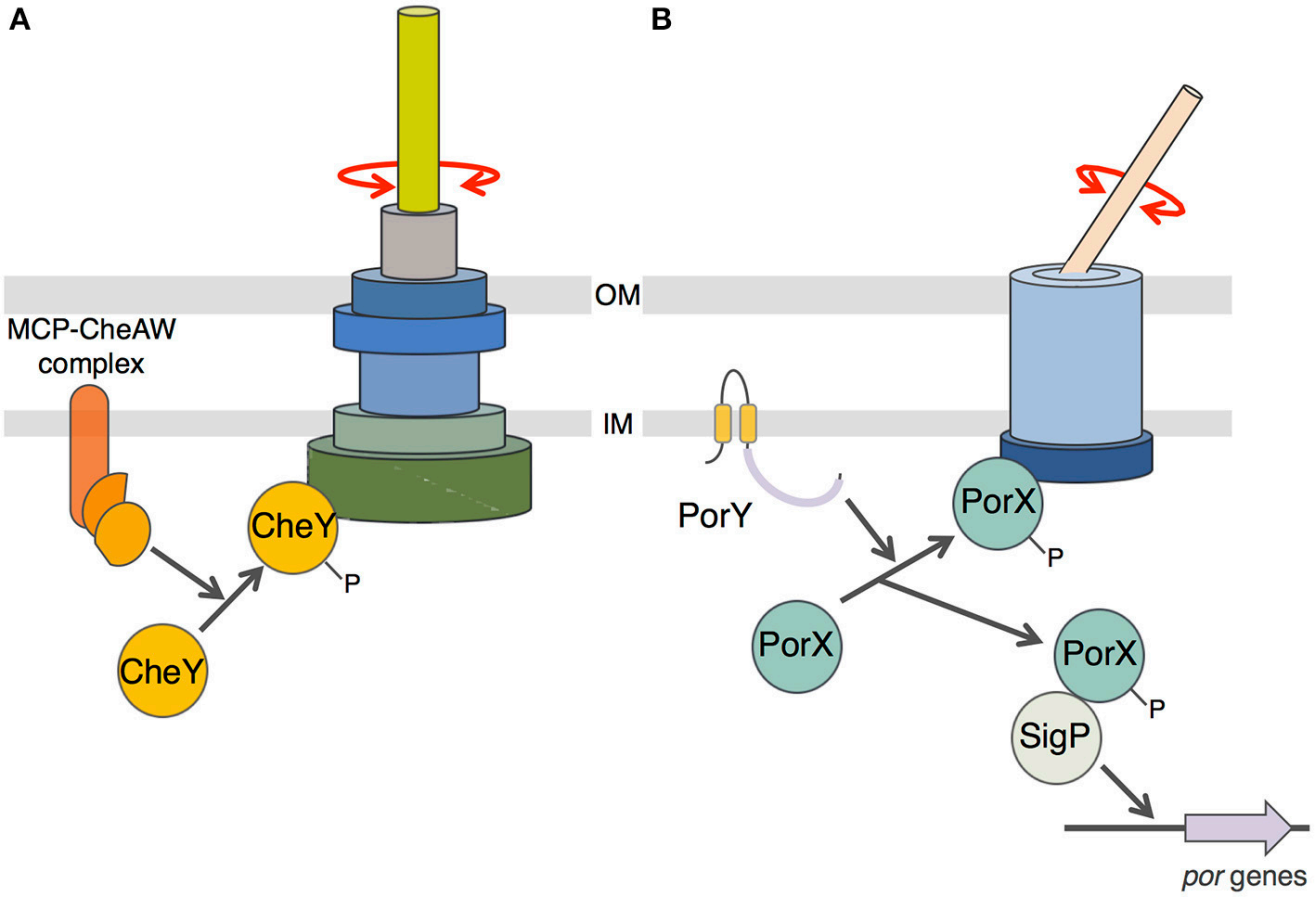
**Schematic comparison between flagellum and T9SS rotary mechanisms. (A)** CheY-dependent chemotaxis-flagellum rotation coupling. Phosphorylation of the CheY protein by the MCP/CheA/CheW complex induces recruitment of phospho-CheY to the C-ring switch complex and reversal of the flagellum filament rotation. **(B)** Putative model of PorX function. Phosphorylation of PorX protein by PorY induces (i) PorX-SigP complex formation and stabilization of the SigP sigma factor involved in *por* genes regulation, and (ii) recruitment of phospho-PorX to the T9SS complex and reversal of the cell surface filament adhesin rotation.

Finally, we showed that PorX also interacts with the cytoplasmic domain of an inner membrane component of the T9SS, PorL. This interaction is mediated by a hydrophobic patch located at the C-terminal extremity of PorL. This behavior is reminiscent of the interaction of CheY with the switch complex that constitutes the basal C-ring structure of the flagellum (Roman et al., 1992; Shukla et al., 1998; Sagi et al., 2003; Paul et al., 2006; Sarkar et al., 2010; Figure 5A). CheY binding on the C-ring induces the reversal of flagellum rotation (Roman et al., 1992; Sourjik and Wingreen, 2012). Based on (i) the homology of the receiver domain of PorX with CheY, (ii) its interaction with PorL, and (iii) the recent observation that the *F. johnsioniae* T9SS is a rotary machine (Nakane et al., 2013; Shrivastava and Berg, 2015; Shrivastava et al., 2015), we propose a working model in which phosphorylated PorX binds to PorL, and similarly to CheY, induces T9SS rotation reversal (Figure 5B). It would be interesting to test this hypothetical model in *F. johnsioniae* by following T9SS rotation and by tracking the gliding activity of *porX* mutant cells. Understanding how the T9SS activity is controlled would provide insights for the design of drugs that interfere with T9SS function and for alternative therapeutic treatments for individuals suffering of gum diseases.

## Author contributions

MV and EC designed the study. MV and ED performed the experiments. EC wrote the manuscript.

### Conflict of interest statement

The authors declare that the research was conducted in the absence of any commercial or financial relationships that could be construed as a potential conflict of interest.
