# Supplementary material for: The PorX Response Regulator of the Porphyromonas gingivalis PorXY Two-Component System Does Not Directly Regulate the Type IX Secretion Genes but Binds the PorL Subunit
Source: Front Cell Infect Microbiol. 2016 Aug 31;6:96. doi: 10.3389/fcimb.2016.00096 (PMC5005315; doi:10.3389/fcimb.2016.00096)
Supplement: Supplementary file 1 [file Table1.PDF]

Strains, plasmids and oligonucleotides used in this study.

Strains

Strains	Description and genotype	Source
E. coli K-12		
DH5α	F-, Δ(*argF-lac*)U169, *phoA*, *supE44*, Δ(lacZ)M15, *relA*, *endA*, *thi*, *hsdR*	New England Biolabs
W3110	F-, lambda- IN(*rrnD-rrnE*)1 *rph*-1	Laboratory collection
BTH101	F-, *cya-99, araD139, galE15, galK16, rpsL1 (Str r), hsdR2, mcrA1, mcrB1.*	Karimova *et al.*, 1998
BL21(DE3)	*fhuA2 [lon] ompT gal (λ DE3) [dcm] ΔhsdS λ DE3 = λ sBamHIo ΔEcoRI-B int::(lacI::PlacUV5::T7 gene1) i21 Δnin5*	Laboratory collection
P. gingivalis		
ATCC 33277	Wild-type	DSMZ

Plasmids

Vectors	Description	Source
Expression vectors		
pBAD24	cloning vector, pBR322 origin, P*ara*, *araC*, Amp^R	Guzman *et al.*, 1995
pBAD24-$PorX_{VSV-G}$	*porX* cloned into pBAD24, C-terminal VSV-G epitope	This study
pASK-IBA37(+)	cloning vector, P*tet*, Amp^R	IBA Technology
pASK-IBA37-$PorL_{FL}$	*porL* cloned into pASK-IBA37, N-terminal FLAG epitope	This study
Vectors for protein purification		
pETG20A	P*T7*, TRX-6×His-TEV sequence, Amp^R	Arie Geerlof (Munich, Germany)
pETG20A-PorX	*porX* cloned into pETG20A, N-terminal TRX-6×His-TEV sequence	This study
pRSF	P*T7*, 6×His, Kan^R	Novagen
pRSF-$PorY_C$	*porY* residues 173-395 cloned into pRSF, N-terminal 6×His	This study

Bacterial two-hybrid vectors		
pT18-FLAG	Bacterial two-hybrid vector, ColE1 origin, P*lac*, T18 fragment of *Bordetella pertussis* CyaA, Amp^R, FLAG epitope	Battesti and Bouveret, 2008
pT18-PorX	*porX* cloned downstream the T18 coding sequence into pUT18-FLAG	This study
pT18-Hcp	*hcp* cloned upstream the T18 coding sequence into pUT18-FLAG	Zoued *et al.*, 2013
pT18-Pal	*pal* cloned downstream the T18 coding sequence into pUT18-FLAG	Battesti and Bouveret, 2008
pKT25	Bacterial two-hybrid vector, p15A origin, P*lac*, T25 fragment of *Bordetella pertussis* CyaA, Kan^R	Karimova *et al.*, 1998
pT25-FLAG	Bacterial two-hybrid vector, p15A origin, P*lac*, T25 fragment of *Bordetella pertussis* CyaA, Kan^R, FLAG epitope	Battesti and Bouveret, 2008
pT25-$PorY_C$	*porY* residues 173-395 cloned downstream the T25 coding sequence into pT25-FLAG	This study
pT25-Hcp	*hcp* cloned upstream the T25 coding sequence into pT25-FLAG	Zoued *et al.*, 2013
pT25-PorL	*porL* cloned downstream the T25 coding sequence into pKT25	This study
pT25-$PorL_C$	*porL* residues 73-309 cloned downstream the T25 coding sequence into pKT25	This study
pT25-$PorL_{C\Delta Ct}$	*porL* residues 73-274 cloned dpwnstream the T25 coding sequence into pKT25	This study
pT25-porM	*porM* cloned downstream the T25 coding sequence into pKT25	This study
pTolB-T25	*tolB* cloned upstream the T25 coding sequence in pT25-FLAG	Battesti and Bouveret, 2008
Vectors for translational fusion to *gfp*		
pUA66	*gfp,* Kan^R	Zaslaver *et al.,* 2006
pUA66-P*sov*	406-bp upstream *sov* ATG (-373 to +33) cloned upstream the *gfp* sequence in pUA66	This study
pUA66-P*porT*	473-bp upstream *porT* ATG (-413 to +60) cloned upstream the *gfp* sequence in pUA66	This study
pUA66-P*porP*	343-bp upstream *porP* ATG (-309 to +34) cloned upstream the *gfp* sequence in pUA66	This study
pUA66-P*sci1*	EAEC T6SS *sci1* promoter cloned upstream the *gfp* sequence in pUA66	This study

Oligonucleotides

**Name**	**Destination**	**Sequence (5' ➔ 3')**
For plasmid construction [a]		
5-PorX-pBAD	insertion of $porX_{VSVG}$ into pBAD24	CTCTCTACTGTTTCTCCATACCCGTTTTTTTGGGCTAGCAGGAGGTATTACACCATGGAAAAAAACATGAGACCGTATACCGTACTATG
3-PorX-pBAD	insertion of $porX_{VSVG}$ into pBAD24	GGTCGACTCTAGAGGATCCCCGGGTACCTTA**TTTTCCTAATCTATTCATTTCAATATCTGTATA**CTTGGGTTGCATCGTAATTACGGGC
5-T18-PorX	insertion of *porX* downstream T18	GCGGATAACAATTTCACACAGGAAACAGCTATGGAAAAAAACATGAGACCGTATACCGTACTATG

3-T18-PorX	insertion of *porX* downstream T18	GCATGCAAGCTTGGCGTAATCATGGTCTTGGGTTGCATCGTAATTACGGGC
5-T25-PorY_{C}	insertion of *porY* residues 173-395 downstream T25	GACTCTAGAGGATCCCCGGGTACCGCTGAGCGCAGATCCGAACAGAATC
3-T25-PorY_{C}	insertion of *porY* residues 173-395 downstream T25	GTAAAACGACGGCCAGTGAATTCTTACTTACTTATGGCAGATCGGTCTGTT TCGGC
5-T25-PorL	insertion of *porL* downstream T25	GAAG*TCTAGA*TGGTCATTATAGAAGATACAAGAACATCCTTGAG
5-T25-PorL_{C}	insertion of *porLc* residues 73-309 downstream T25	GAAG*TCTAGA*TGCAATGGAATATCACTGGGAAGAGGTCTTC
3-T25-PorL	insertion of *porL* downstream T25	GAAG*GGTACC*CCTAAGGGTGAGCTGCCGGATGATGAAG
3-T25-$\mathrm{PorL}_{C\Delta Ct}$	insertion of *porLc* residues 73-274 downstream T25	GAAG*GGTACC*CCCTCGTTGAGTTGGGTCAGTTGGCG
5-T25-PorM	insertion of *porM* downstream T25	GAAG*TCTAGA*TGCAGTAGGTTCTAATGGGAATGCCAATAG
3-T25-PorM	insertion of *porM* downstream T25	GAAG*GGTACC*CCGTTCACAATTACTTCAATGGCCGGAATCTTAC
5-pIBA37-PorL-FL	insertion of *porL* into pASK-IBA37(+)	GACAAAAATCTAGAAATAATTTTGTTTAACTTTAAGAAGGAGATATACAA ATG**GATTATAAAGATGACGATGACAAG**GGTCATTATAGAAGAT ACAAGAACATCCTTGAG
3-pIBA37-PorL	insertion of *porL* into pASK-IBA37(+)	GATGGTGATGGTGATGCGATCCTCTGCTAGCTTATAAGGGTGAGCTGCCG GATGATGAAG
PorX_p17tev-F	insertion of *porX* into pETG20A	GGGGACAAGTTTGTACAAAAAACCAGGCTTAGAAAACCTGTACTTCCAGG GTGAAAAAAACATGAGACCGTATACCG
PorX-pT17-R	insertion of *porX* into pETG20A	GGGGACCACTTTGTACAAGAAAGCTGGGTTTATTACTTGGGTTGCATCGTA ATTACGGGC
5-*PromSov*-UA66	insertion of *Psov* upstream *gfp*	GTCTTCACCTCGAGGGGATCCTACGACGGCACGGCTCATCC
3-*PromSov*-UA66	insertion of *Psov* upstream *gfp*	GTTCTTCTCCTTTACTCATATGTATATCTCCTTCTTAAATCTAGATATCAAG ACACCCGTGATCCTTCTTTGTTTG
5-*PromPorT*-UA66	insertion of *PporT* upstream *gfp*	GTCTTCACCTCGAGGGGATCCCAGGCGGTCCTTATACTTCCTGTAGGC
3-*PromPorT*-UA66	insertion of *PporT* upstream *gfp*	GTTCTTCTCCTTTACTCATATGTATATCTCCTTCTTAAATCTAGACTGAGAG AATAATCTTCAATTCTTTATTTGGTGTGTAAGATC
5-*PromPorP*-UA66	insertion of *PporP* upstream *gfp*	GTCTTCACCTCGAGGGGATCCGTGCTAAGTATTATTGGACAAAAAACTCT AACCATTATGTACTG
3-*PromPorP*-UA66	insertion of *PporP* upstream *gfp*	GTTCTTCTCCTTTACTCATATGTATATCTCCTTCTTAAATCTAGAGACTGAT GCCACGGTGGTAAAACGAAG
5-*PromSci1*-UA66	insertion of *Psci1* upstream *gfp*	GTCTTCACCTCGAGGGGATCCTGTTGGGCCCTTCCTCCATATCTTTCC
3-*PromSci1*-UA66	insertion of *Psci1* upstream *gfp*	GTTCTTCTCCTTTACTCATATGTATATCTCCTTCTTAAATCTAGAGGCTCTC TTCCTGTGAAACCTGCATC
5-pRSF-PorY_{C}	insertion of *porY* residues 173-395 into pRSF	CATCATCACCACAGCCAGGATCCGGCTGAGCGCAGATCCGAACAGAATCG
3-pRSF-PorY_{C}	insertion of *porY* residues 173-395 into pRSF	GGCGCGCCGAGCTCGAATTTATGGCAGATCGGTCTGTTTCGGC

[a] Sequence annealing on the target plasmid underlined.
[b] Restriction site *italicized*.
[c] FLAG or VSV-G epitope coding sequence in **Bold.**
